# Sesamolin Protects Mice From Ovariectomized Bone Loss by Inhibiting Osteoclastogenesis and RANKL-Mediated NF-κB and MAPK Signaling Pathways

**DOI:** 10.3389/fphar.2021.664697

**Published:** 2021-06-14

**Authors:** Xue Yang, Jiamin Liang, Ziyi Wang, Yuangang Su, Yunfei Zhan, Zuoxing Wu, Jing Li, Xuedong Li, Runfeng Chen, Jinmin Zhao, Jiake Xu, Qian Liu, Bo Zhou

**Affiliations:** ^1^ Guangxi Key Laboratory of Regenerative Medicine, Guangxi-ASEAN Collaborative Innovation Center for Major Disease Prevention and Treatment, Guangxi Medical University, Guangxi, China; ^2^ School of Biomedical Sciences, The University of WA, Perth, WA, Australia; ^3^ Jiu Jiang No. 1 People’s Hospital, Jiangxi, China; ^4^ Department of Nuclear Medicine, School of Medicine, Zhongshan Hospital, Xiamen University, Fujian, China; ^5^ Research Centre for Regenerative Medicine, Orthopaedic Department, The First Affiliated Hospital of Guangxi Medical University, Guangxi, China

**Keywords:** sesamolin, NF-κB, MAPK, osteoclast, postmenopausal osteoporosis

## Abstract

This article was submitted to Experimental Pharmacology and Drug Discovery, a section of the journal Frontiers in Pharmacology. Postmenopausal osteoporosis (PMOP), which increases the risk of fracture, is the most common bone disease in women. PMOP not only increases the risk of death but also imposes a financial burden on countless families. At present, most of the drugs used to treat osteoporosis have significant side effects, so it is important to find effective anti-osteoporosis medications without major side effects. Sesamolin (Ses) is a kind of natural lignan extracted from sesame oil. Many researches have shown that Ses has anti-inflammatory, antioxidative, and anticancer effects, however it is still unknown whether it has any effect on osteoporosis. In this research, we explored the therapeutic effect of Ses in the process of osteoclast formation and bone resorption and found that Ses effectively inhibited osteoclast formation *in vitro* through TRAcP staining and hydroxyapatite resorption assays. Through Western blot analysis of the NF-κB pathway, MAPK pathway, c-Fos and NFATc1, it was found that Ses not only effectively inhibited the activation of NF-κB and MAPK signaling pathways induced by RANKL but also significantly reduced the protein expression of c-Fos and NFATc1. Several genes specifically expressed in osteoclasts were determined by qPCR, and Ses was also found to play a significant inhibitory role on the expression of these genes. Besides, an osteoporosis model induced in ovariectomized (OVX) mice was employed to verify that Ses could effectively reduce bone loss caused by estrogen deficiency *in vivo*. In conclusion, Ses showed promise as a new treatment for postmenopausal osteoporosis.

## Introduction

Osteoporosis is a systemic bone disease with global public health effects, and it most commonly affects postmenopausal women. Due to decreases in bone strength, bone brittleness results in an increase in the risk of fracture (Author Anonymous, 1993; Svedbom et al., 2013; Noel et al., 2018). Postmenopausal osteoporosis (PMOP) not only increases mortality but also imposes a financial burden on countless families. Most deaths caused by osteoporotic fractures in the United States occur in people over the age of 75; in 2003, the cost of treatment for osteoporotic fractures was as high as $17 billion, and is expected to increase to $25 billion by 2025 (Cheng et al., 2009). In 2017, the total direct cost of osteoporosis caused by osteopenia and fractures in Australia was as high as US $2.77 billion; fracture treatment accounted for 68% of this spending, and the most common recipients of treatment for fractures were women aged 70 and over (Tatangelo et al., 2019). At present, there are two main types of drugs used for the treatment of osteoporosis: anti-resorption drugs that slow bone resorption, and pro-anabolic drugs that promote bone formation. Many of these drugs are not ideal, because most of them have significant side effects, such as the bisphosphonate as an anti-resorption drug that can cause mandibular necrosis and esophageal irritation; raloxifene, which can cause thromboembolic disease; teriparatide, which can cause hypercalcemia (Rachner et al., 2011). Therefore, we urgently need to find new drugs for the treatment of osteoporosis to improve the limitations of the current therapeutic options.

The key factors for maintaining bone homeostasis are closely related to the coordination between bone resorption by osteoclasts and bone formation by osteoblasts (Sims and Martin, 2020). Estrogen deficiency is an important part of the pathogenesis of PMOP. Estrogen reduces the rate of bone remodeling by inhibiting osteoclasts and bone resorption (Manolagas et al., 2013; Xiong et al., 2015; Li et al., 2020). Osteoprotegerin (OPG) and receptor activator of nuclear factor kappa B (NF-κB) ligand (RANKL) are an essential parts in the signal transduction in bone homeostasis (Kim et al., 2007; Piri et al., 2016). The cytokines OPG and RANKL are important factors via affecting osteoclast formation and osteoblast activity. An increase in estrogen leads to elevation of the cytokine OPG secreted by osteoblasts; and reduction of secretion of the cytokine RANKL expressed on the surface of osteoblasts, thus affecting osteoclast formation and bone resorption, resulting in an increase in bone mineral density (Kim et al., 2007; Piri et al., 2016). RANKL and macrophage-colony stimulating factor (M-CSF), are two important cytokines regulating the differentiation and formation of multinucleated osteoclasts derived from bone marrow macrophages (BMMs) (Suda et al., 1992; Nakagawa et al., 1998; Arai et al., 1999). RANKL induces activation of multiple early signal transduction pathways, such as the NF-κB and mitogen-activated protein kinase (MAPK) pathways, which eventually leads to the activation of c-Fos and nuclear factor of activated T-cells 1 (NFATc1) (Shinohara and Takayanagi, 2007; Honma et al., 2020).

High-quality oil accounts for 50–60% of the composition of sesame, and lignans are common compounds in sesame that have a variety of health-promoting effects (Pathak et al., 2014). At present, lignans are known to have anti-inflammatory, antioxidative, antiaging and anticancer properties (Wu M.-S. et al., 2019). Sesamolin (Ses) is a major lignan extracted from sesame oil that has a variety of biological activities (Wu D. et al., 2019; Michailidis et al., 2019; Baek et al., 2020). According to current reports, we found that there may be a significant correlation between Ses and MAPK signal pathway. At present, studies have shown that Ses increases the cell dissolution activity of natural killer (NK) cells through the MAPK signaling pathway and induces a strong killing activity of NK cells (Lee and Lee, 2018). In addition, it was found that Ses could inhibit the activation of p38 MAPK and protect microglia from H_2_O_2_-induced cell damage (Hou et al., 2005). MAPK signal pathway is the early signal transduction pathway downstream of RANKL, and RANKL is the key factor affecting osteoclast formation (Shinohara and Takayanagi, 2007; Honma et al., 2020). We speculated that Ses might be related to osteoclast formation.

We conducted a preliminary exploration to ascertain effects of compounds on osteoclastogenesis by TRAcP staining, and we found that Ses can indeed inhibit the differentiation of osteoclasts *in vitro*, and still has no obvious toxic effect on the proliferation of osteoclasts up to 40 μM. We further hypothesized that Ses might inhibit osteoclasts through the RANKL-induced MAPK and NF-κB signal transduction pathways to prevent the bone loss caused by osteoporosis. The uppermost objective of this research was to investigate the therapeutic effect of Ses on osteoclast formation and bone resorption *in vitro* as well as in a model of osteoporosis caused by ovariectomy *in vivo*.

## Materials and Methods

### Reagents and Materials

Ses was purchased from Chengdu DeSiTe Biological Technology Co., Ltd. (Sichuan, Chengdu, China, #CAS 526-07-8), and its purity was over 98%. Ses was dissolved into a concentration of 100 mM with dimethyl sulfoxide (DMSO) and further diluted to the working concentration with alpha-modified minimal essential medium (α-MEM). Estrogen (E_2_) and Cell Counting Kit-8 (CCK-8) were obtained from Med Chem Express (Monmouth, New Jersey, United States). α-MEM, Penicillin-streptomycin solution and fetal bovine serum (FBS) were purchased from Gibco (Thermo Fisher Scientific, Waltham, Massachusetts, United States). The primary antibodies (included phosphorylated as well as total forms of p65 (# 3033S and 8242S), ERK (# 4370S and 4695S), JNK (# 4668S and 9252S) and p38 (# 4511S and 8690S); β-actin (# 4970S), IκB-α (# 4812S)) and secondary antibodies (included rabbit and mouse) were obtained from Cell Signaling Technology (Danvers, MA, United States). The primary antibodies against NFATc1 and c-Fos were obtained from Santa Cruz Biotechnology (Dallas, CA, United States, # sc7294) and Abcam (Cambridge, United Kingdom, # ab134122), respectively.

### Osteoclast Culture *In Vitro*

With the approval of the Animal Ethics Committee of Guangxi Medical University (Nanning, Guangxi, China), C57BL/6J mice were purchased, and used to isolate the bone marrow macrophages (BMMs). After washing out the bone marrow with a 1 ml syringe in a sterile environment, BMMs were then cultured in the necessary α-MEM culture (supplemented with 10% FBS, 1% penicillin-streptomycin and 25 ng/ml M-CSF). On the third day, the culture medium was changed once, and finally the attached cells were used in the follow-up experiment.

BMMs were cultured with 96-well plates, and incubated overnight in the incubator. After allowing the cells to adhere to the bottom, BMMs were induced by RANKL stimulation with or without Ses (0, 2.5, 5, 10 μM), and the culture medium was changed every 2 days for more than 5 days until osteoclasts formed. To further explore the time dependence of Ses on the differentiation and formation of osteoclasts, BMMs were treated with 10 μM Ses at three stages (early: day 1 to day 3, middle: day 3 to day 5, late: day 5 to day 7) under RANKL stimulation, and the cells that were not treated with Ses by RANKL stimulation were used as a positive control. The cells were fixed with 4% paraformaldehyde (PFA) solution for 15 min, subsequently stained with tartrate-resistant acid phosphatase (TRAcP) reagent to visualize enzyme activity. Cytation 5 (BioTek Instruments Inc., Winooski, VT, United States) was used to take staining pictures, and cells with at least 3 nuclei were classified as osteoclasts and counted.

### Cytotoxicity Assay

BMMs were cultured with 96-well plates, and incubated overnight in the incubator. After allowing the cells to adhere to the bottom, BMMs were treated with or without Ses (0, 1.25, 2.5, 5, 10, 20, 40 μM), and CCK-8 reagent was added after 2 days, followed by incubation in the incubator for 2 h. A TriStar^2^ LB 942 Multimode Microplate Reader (Berthold Technologies Gmbh & Co. KG, Baden-Württemberg, Germany) was used to detect the liquid absorbance value (OD) at 450 nm.

### Podosomal Actin Belt Immunofluorescence

To investigate whether Ses can affect the actin cytoskeleton in osteoclasts, BMMs were induced by RANKL stimulation with or without Ses (0, 5, 10 μM). Afterwards, 4% PFA was used to fix the cells for 15 min, subsequently washed three times with an appropriate amount of PBS. 0.1% Triton X-100 was used to incubate the cells for 5 min at room temperature, and sealed with 3% BSA in PBS for 30 min. After that, the samples were transferred to the dark condition and dyed with rhodamine phalloidin for 1 h. Afterward, the cells were washed with proper amount of PBS and stained with DAPI for 5 min, washed again with proper amount of PBS, and finally photographed with Cytation 5.

### Detection of Hydroxyapatite Absorption *In Vitro*

BMMs were inoculated into a 6-well plate, and the number of the cells were 1 × 10^5^ per well. After allowing the cells to adhere to the bottom overnight, they were induced by RANKL until small osteoclasts began to form. Then cells were collected, and inoculated into an OsteoAssay plate (Corning Inc., Corning, NY, United States) coated with hydroxyapatite at the same density. On the second day, the cells were treated with or without Ses (0, 5, 10 μM). After 48 h, the cells were removed with sodium hypochlorite, the resorption area of hydroxyapatite was observed by Cytation 5, and the whole plate was photographed. Finally, ImageJ software was used to quantify the percentage of the resorption area by the osteoclasts in each well.

### Real-Time Quantitative PCR (qPCR)

BMMs were inoculated into a 6-well plate, and they were induced by RANKL with or without Ses (0, 5, 10 μM) until osteoclasts were observed. Ground the mouse forelimbs under the condition of 60 Hz for 60 s. Total RNA was isolated and extracted by TRIzol (Thermo Fisher Scientific), and then reverse-transcribed into complementary DNA (cDNA) by reverse transcription. Next, the obtained cDNA was used as a template for real-time quantitative PCR on the LightCycler ®96 system (Roche, Basel, Switzerland). The primer sequences of the osteoclast-related genes (f is forward, r is reverse) were as follows: *Ctsk* (f, 5′-AGG​CGG​CTC​TAT​ATG​ACC​ACT​G-3′; r, 5′-TCT​TCA​GGG​CTT​TCT​CGT​TC-3′), *Mmp-9* (f, 5′-GAA​GGC​AAA​CCC​TGT​GTG​TGT​T-3′; r, 5′-AGA​GTA​CTG​CTT​GCC​CAG​GA-3′), *Dc-stamp* (f, 5′-TCT​GCT​GTA​TCG​GCT​CAT​CTC-3ʹ; r, 5′-ACT​CCT​TGG​GTT​CCT​TGC​TT-3ʹ), *NFATc1* (f, 5ʹ-GGT​GCT​GTC​TGG​CCA​TAA​CT-3ʹ; r, 5ʹ-GAA​ACG​CTG​GTA​CTG​GCT​TC-3ʹ), *c-Fos* (f, CCA​GTC​AAG​AGC​ATC​AGC​AA; r, AAG​TAG​TGC​AGC​CCG​GAG​TA), *Gapdh* (f, 5ʹ-AAC​TTT​GGC​ATT​GTG​GAA​GG-3ʹ; r, 5ʹ-ACA​CAT​TGG​GGG​TAG​GAA​CA-3ʹ). The reaction conditions of PCR amplification were: denaturation at 95°C for 10 min and followed by 55 cycles at the same temperature for 15 s, then decreased to 60°C for 15 s, and finally increased to 72°C for 40 s. For analysis, the 2^−ΔΔCT^ method was used and the target gene expressions were normalized to *Gapdh*.

### Western Blot

To study the early effect of Ses on osteoclast activation-related signaling pathways, BMMs were inoculated in a 6-well plate, and the number of the cells were 1 × 10^6^ cells per well. After allowing the cells to adhere to the bottom overnight, the control group and the drug group were starved with serum-free medium for 3 h, while the drug group was treated with 10 μM Ses, and then induced with 100 ng/ml RANKL for 0, 5, 10, 20, 30 or 60 min. To study the late effect of Ses on osteoclast activation-related signaling pathways, BMMs were inoculated in a 6-well plate, and the number of the cells were 1 × 10^5^ cells per well, subsequently the cells were allowed to attach overnight to the orifice plate. 100 ng/ml RANKL was used to stimulated the control and drug groups (10 μM Ses) for 0, 1, 3, and 5 days. Then, radioimmunoprecipitation (RIPA) was used to split the cells and extract proteins. Afterwards, the proteins by sodium dodecyl sulphate-polyacrylamide gel electrophoresis (SDS-PAGE) and then transferred to nitrocellulose membranes (Thermo Fisher Scientific, Shanghai, China). Then, the nonspecific immunoreactivities were blocked by incubating the membranes in 5% skim milk for 1 h. Then the membranes were incubated with primary antibodies and shaken gently at 4°C for 12 h. On the second day, after washing the membranes three times, the membranes were incubated with the corresponding secondary antibodies at room temperature for 1 h. Images were obtained using the ImageQuant LAS-4000 system (GE Healthcare, Chicago, Illinois, United States) and were finally analyzed by ImageJ software.

### Construction of Animal Model *In Vivo*

The use of OVX-induced osteoporosis model in mice and all subsequent experimental protocols were approved by the Ethics Committee of Guangxi Medical University. First, we randomly divided twenty-four C57BL/6J mice (female, 11-week-old) into four groups (*n* = 6 mice in each group): Sham group, OVX + Vehicle group, OVX + 100 ng/kg E_2_ group and OVX + 5 mg/kg Ses group. Under tribromoethanol anesthesia, the groups were separately subjected to sham surgery without ovariectomy or bilateral ovariectomy to induce osteoporosis. Seven days after the operation, the mice in OVX + Ses group were intraperitoneally injected with 5 mg/kg Ses, while the group of Sham and OVX + Vehicle were intraperitoneally injected with 1% DMSO in PBS, and the OVX + E_2_ group was injected intraperitoneally with 100 ng/kg E_2_ as the control for 42 days. Eventually, all the mice were sacrificed, the femurs were extracted, and excess tissue was removed for microscopic computed tomography (micro-CT) and histological evaluation.

### Micro-CT Data Analysis

The left femur was scanned with a SCANCO MEDICAL Micro-CT 50 instrument (SCANCO Medical AG, Switzerland). The main parameters of scanning the femur were as follows: precision/slice thickness 9 µm, voltage 70 kV, current 200 μA, and rotation angle 180°. The region of interest (ROI) was then selected, and 8 mm sections of the ROI of the distal femur were scanned. After the scan was completed, the region of interest (thickness 0.9 mm) was selected for 3D reconstruction and data were analyzed with Mimics 19.0, Magics 19.01 and ABA special bone analysis software. The bone parameters were analyzed as follows: cortical bone thickness (Ct.Th); bone volume fraction (BV/TV); trabecular number (Tb.N), connectivity density (Conn.Dn), thickness (Tb.Th) and spacing (Tb.Sp); bone surface area (BS).

### Histological Analysis

The femur was decalcified with EDTA decalcification solution. The femur, heart, liver, spleen and kidney were dehydrated and embedded in wax. Next, the wax blocks were placed in a microtome and sliced into 4 μm continuous sections. Heart, liver, spleen and kidney sections were stained with hematoxylin and eosin (HE), scanned under a KFPRO scanner (KONFOONG BIOTECH INTERNATIONAL CO., LTD., Ningbo, China), and imaged at 10× and 40× magnification using K-Viewer software. For HE staining, the femur tissue sections were stained with hematoxylin dye for about 4 min, washed with running water, dehydrated with 85 and 95% gradient alcohol, and then stained with eosin dye for 5 min. For TRAcP staining, an appropriate amount of TRAcP staining solution was added and incubated at 37°C for 20 min, while the immunohistochemical detection of Ctsk and OCN were incubated with corresponding antibodies and then stained with Diaminobenzidine (DAB), and finally re-stained with hematoxylin staining solution for 15 s. After staining, the femur tissue sections were scanned under Panoramic DESK, Pmurmidi, P250, P1000 (3D HISTECH, Hungary) scanners. CaseViewer software was used to view and analyze the images at 5× and 20× magnification. The results of TRAcP staining determined that the cytoplasm of osteoclasts was wine red, while the results of Ctsk and OCN expression were judged according to the positive expression of DAB as brown-yellow and the nucleus was light blue.

### Statistical Analysis

Each experiment and data were repeated three times or more, and all the experimental data are expressed as the mean ± standard deviation (SD). The statistical significance was determined by one-way ANOVA or Student’s *t*-test. A *p*-value of less than 0.05 was considered statistically significant.

## Results

### Ses Inhibits Osteoclast Formation *In Vitro*

**FIGURE 1:**
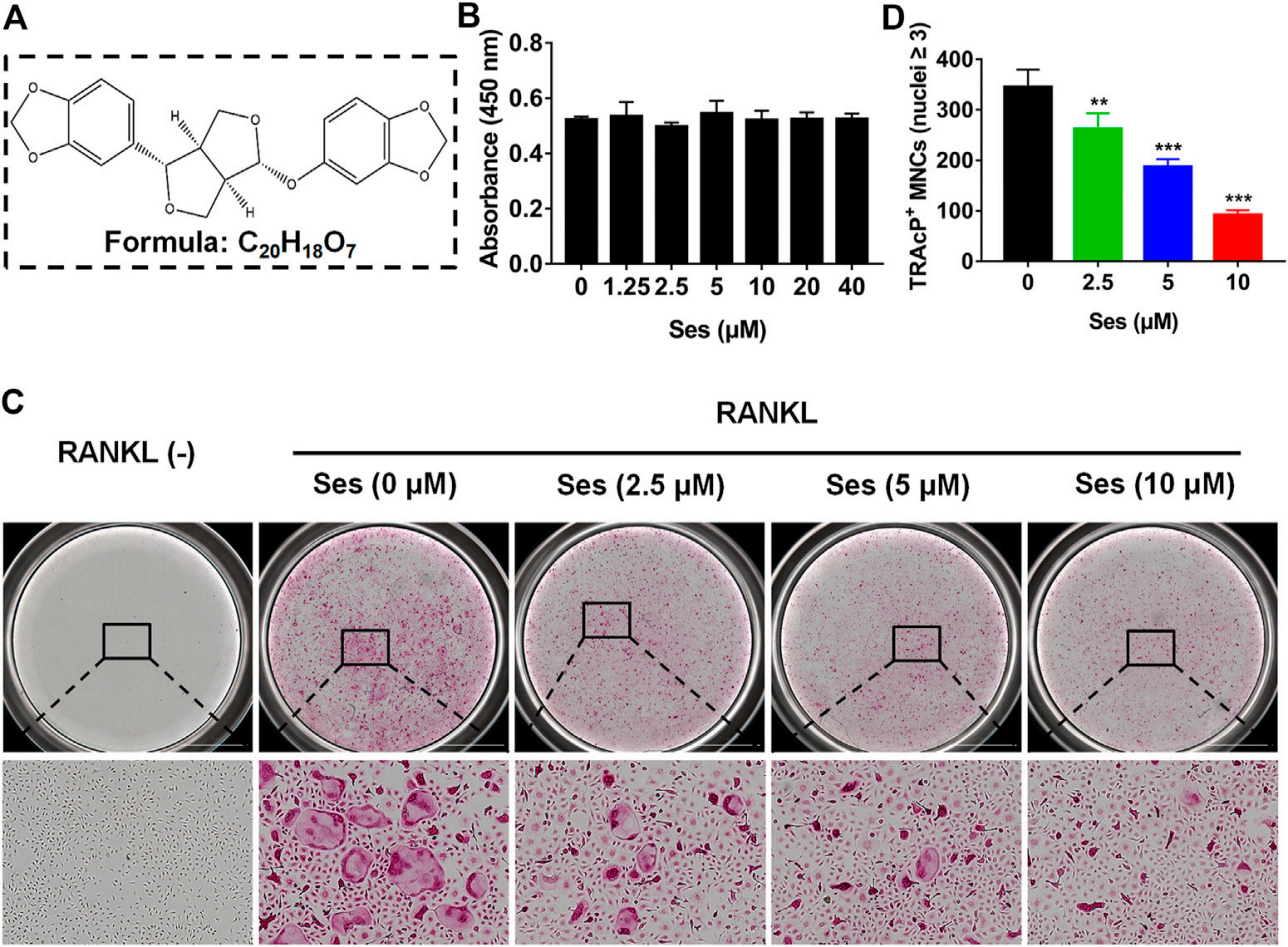
Ses inhibits osteoclast formation induced by RANKL *in vitro*. **(A)** The chemical structure and molecular formula of Ses. **(B)** After 48 h of treatment, the effect of different concentrations of Ses on the viability of BMMs was determined by CCK-8. **(C)** BMMs were stimulated with RANKL for 5 days, and representative images of TRAcP staining showed that Ses inhibited osteoclast formation in a dose-dependent manner (scale = 2,000 μM). **(D)** Quantification of the number of TRAcP-positive multinucleated cells (nuclei ≥3). The above data are expressed as the mean ± SD; *n* = 3; **p < 0.01 and ***p < 0.001. BMMs, bone marrow macrophages; Ses, sesamolin; RANKL, receptor activator of nuclear factor-κB ligand; TRAcP, tartrate-resistant acid phosphatase.

**FIGURE 2:**
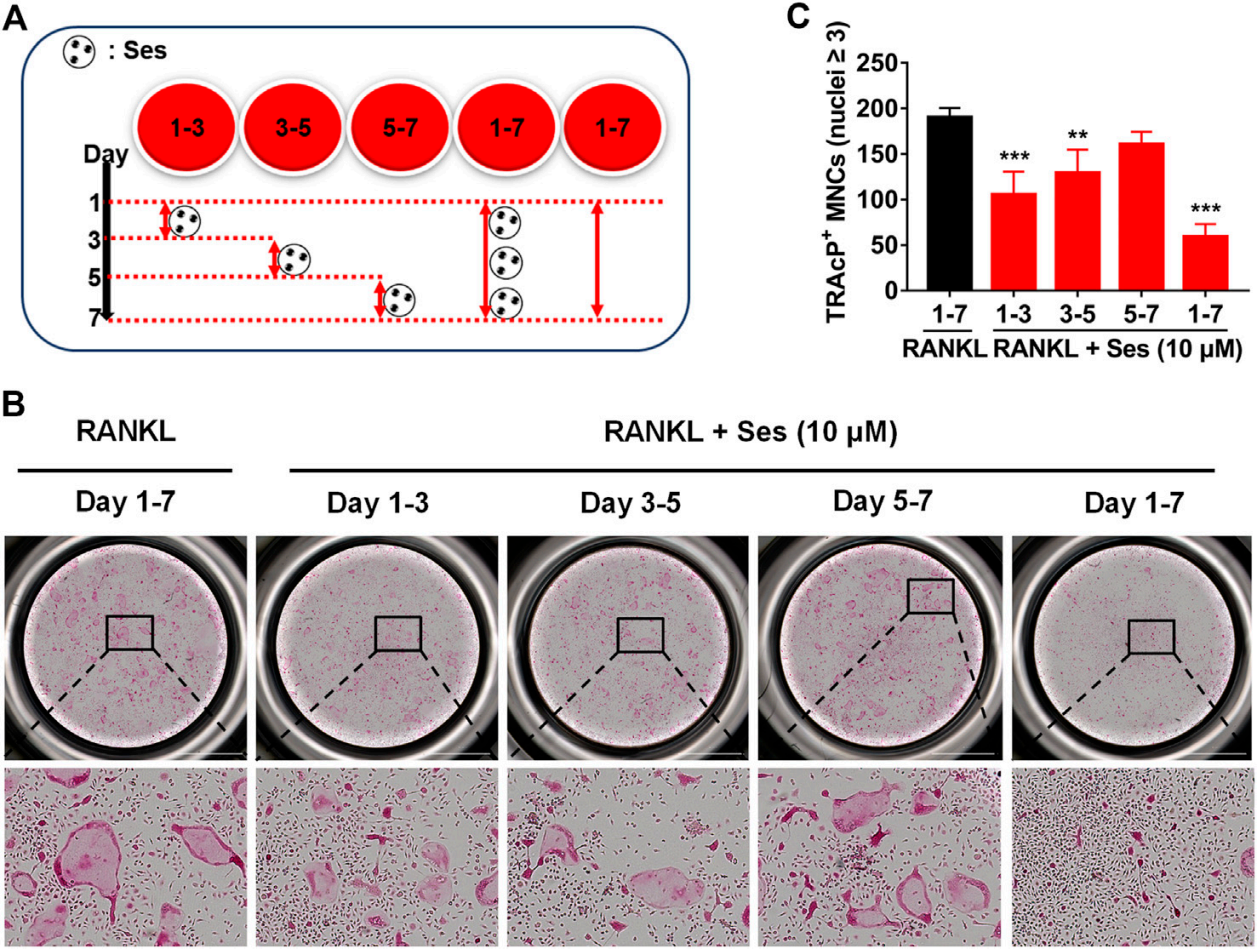
Ses inhibits osteoclast formation induced by RANKL in the early and middle stages. **(A)** Effect of Ses on osteoclast formation in a time-dependent manner. **(B)** The representative image of TRAcP staining showed that Ses (10 μM) was used to treat BMMs within a specified period of time during osteoclast formation (scale = 2,000 μM). **(C)** Quantification of the number of TRAcP-positive multinucleated cells (nuclei ≥3). The above data are expressed as the mean ± SD; *n* = 3; **p < 0.01 and ***p < 0.001. BMMs, bone marrow macrophages; Ses, sesamolin; RANKL, receptor activator of nuclear factor-κB ligand; TRAcP, tartrate-resistant acid phosphatase.

The chemical structure and molecular formula of Ses are shown in Figure 1A. First, we used CCK-8 to evaluate the cell viability and evaluated the possible cytotoxic effect of Ses on BMMs. In this study, when the concentration reached 40 μM, Ses was not found to affect the proliferation of BMMs (Figure 1B). After that, to investigate the influence of Ses in the process of osteoclast formation, BMMs were induced by RANKL with or without Ses (0, 2.5, 5, 10 μM). The control group induced by RANKL (without Ses) resulted in formation of TRAcP-positive multinucleated osteoclasts, while Ses treatment showed dose-dependent inhibition of osteoclast formation (Figures 1C,D). To explore the role of Ses at different stages, Ses (10 μM) was used to treat the cells for the stated times (Figure 2A). We found that Ses mainly inhibited osteoclast differentiation in the early and middle stages (day 1–5), especially in the early stage (day 1–3), but not in the late stage (day 5–7) (Figures 2B,C).

### Ses Affects Podosome Belt Formation in Osteoclasts and Inhibits Osteoclast Resorption

**FIGURE 3:**
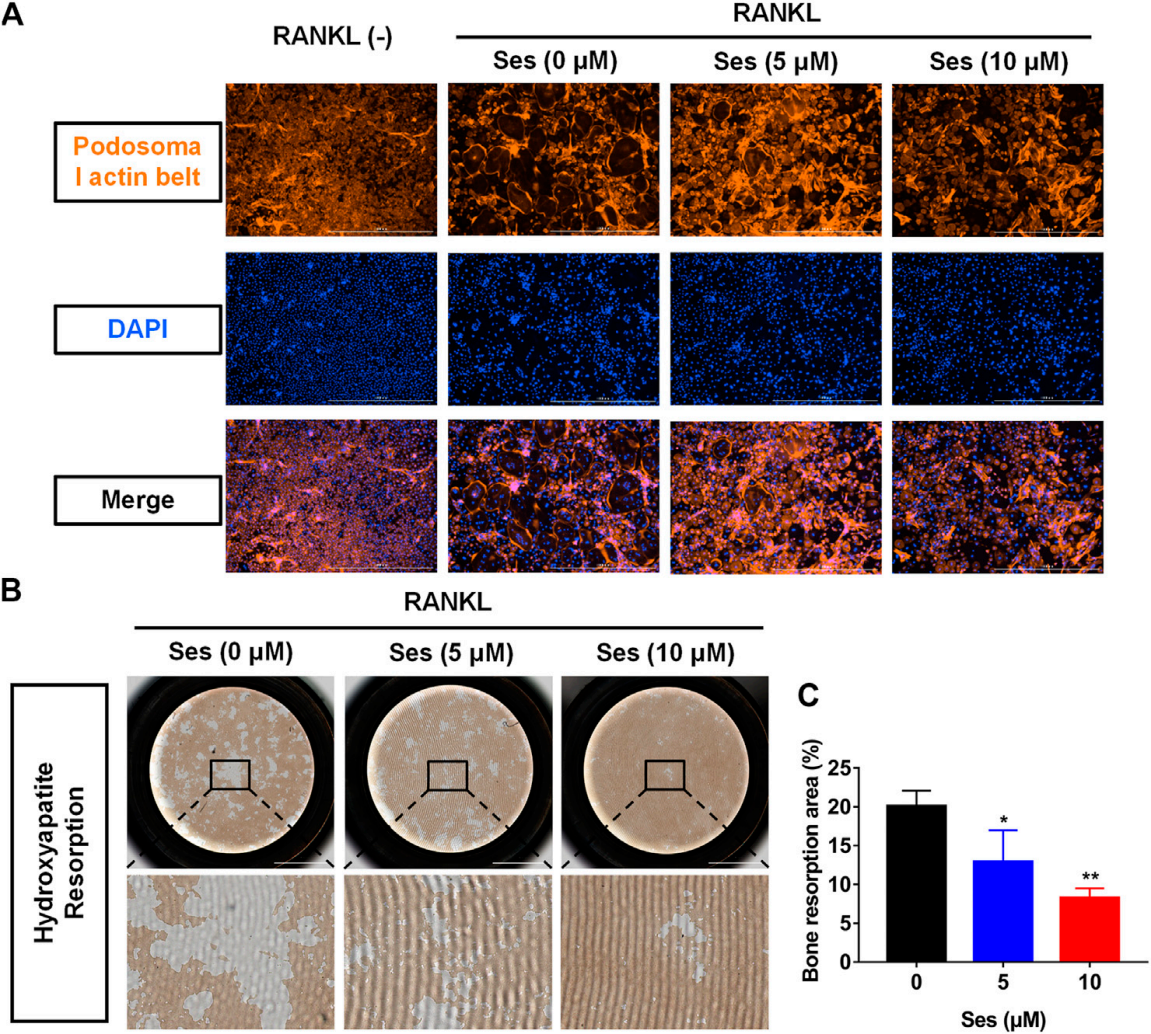
Ses inhibits the bone resorption activity of mature osteoclasts *in vitro*. **(A)** Representative images of podosome belt formation in osteoclasts treated with different concentrations of Ses. The actin cytoskeleton (red) and nucleus (blue) of osteoclasts were stained (scale = 1,000 μM). **(B)** After treatment with Ses, ImageJ was used to analyze the bone resorption area of each group (scale = 2,000 μM). **(C)** Ses inhibited the bone resorption area of osteoclasts induced by RANKL in a dose-dependent manner (scale = 2,000 μM). The above data are expressed as the mean ± SD; *n* = 3; *p < 0.05, **p < 0.01, and ***p < 0.001. Ses, sesamolin; RANKL, receptor activator of nuclear factor-κB ligand.

To observe the influence of Ses on podosome belt formation, cells were stained with rhodamine phalloidin. After RANKL stimulation, a foot band with a complete nucleus was formed around the mature osteoclasts in the absence of Ses. In contrast, the number of osteoclasts and nuclei decreased significantly after treatment with Ses (5 and 10 μM) (Figure 3A).

To explore whether Ses affects osteoclast resorption, we used hydroxyapatite-coated plates to culture osteoclasts. According to the results, the percentage of total resorption area of osteoclasts in the drug group (5, 10 μM Ses) was significantly lower than that in the control group (0 μM Ses) (Figures 3B,C), indicating that Ses could inhibit the bone resorption of osteoclasts.

### Ses Inhibits the Specifically Expressed Genes in Osteoclasts

**FIGURE 4:**
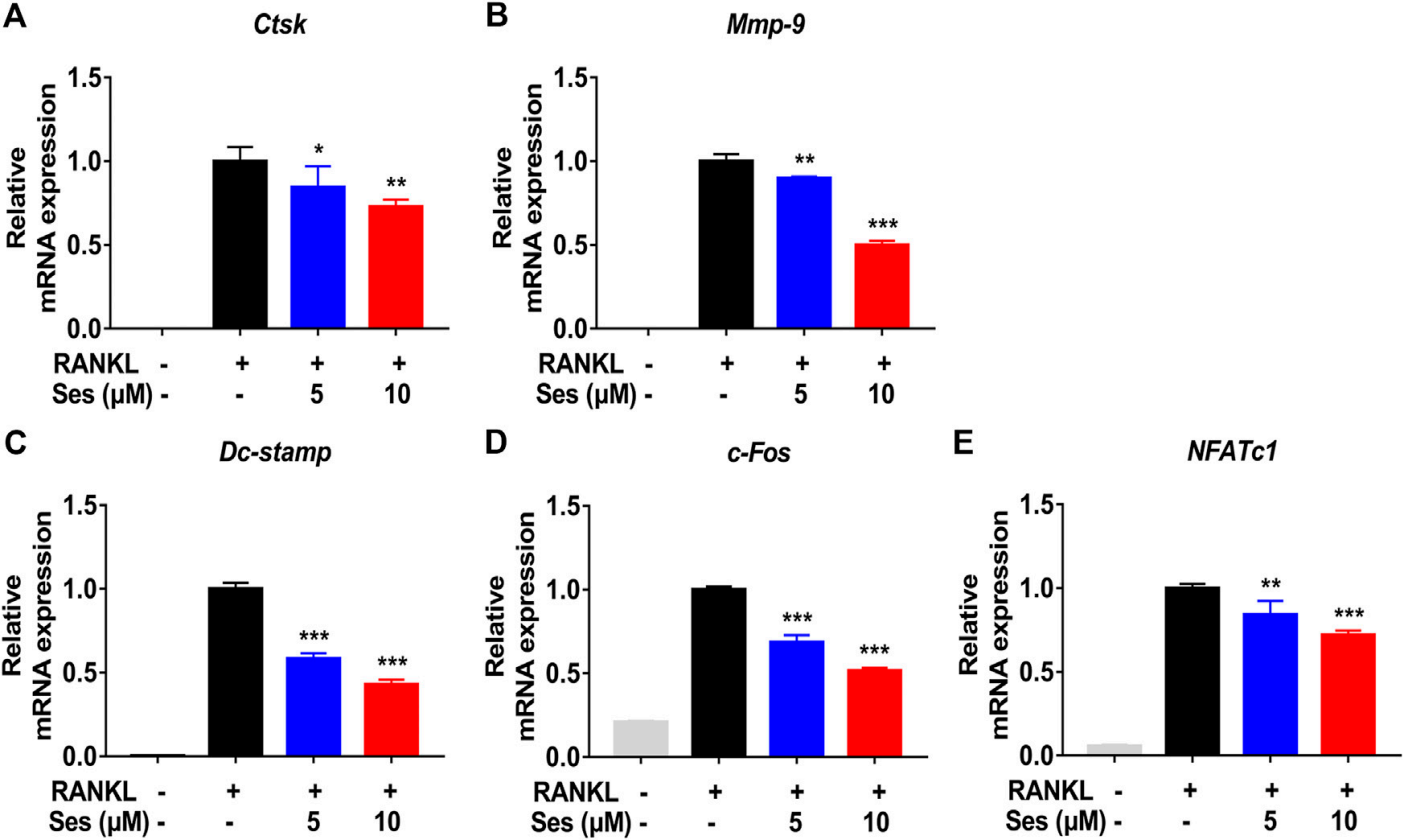
Ses inhibits the expression of osteoclast-specific genes *in vitro*. **(A–E)** The expression levels of the osteoclast-related specific genes *Ctsk*, *Mmp-9*, *Dc-stamp*, *c-Fos* and *NFATc1* were analyzed by qPCR. The above data are expressed as the mean ± SD; *n* = 3; *p < 0.05, **p < 0.01, and ***p < 0.001. Ses, sesamolin; RANKL, receptor activator of nuclear factor-κB ligand; *Ctsk*, cathepsin K; *Mmp-9*, matrix metalloproteinase-9; *Dc-stamp*, dendritic cell-specific transmembrane protein; *c-Fos*, Proto-oncogene C-Fos; *NFATc1*, nuclear factor of activated T cells 1.

*In vitro,* in the process of inducing osteoclast differentiation, several genes specifically expressed in osteoclasts, such as *Ctsk*, *Mmp-9*, *Dc-stamp*, *c-Fos* and *NFATc1*, were down-regulated after treatment with Ses (5, 10 μM) (Figures 4A–E). *In vivo, Ctsk* and *Mmp-9* were also down-regulated after treatment with Ses (5 mg/kg) (Supplementary Figures S1A,B). These results provided further evidence that Ses inhibited the specifically expressed genes in osteoclasts both *in vitro* and *in vivo*, thus inhibiting the formation of osteoclasts.

### Ses Interferes With the Activation of the NF-κB and MAPK Pathways During RANKL-Induced Osteoclast Formation

**FIGURE 5:**
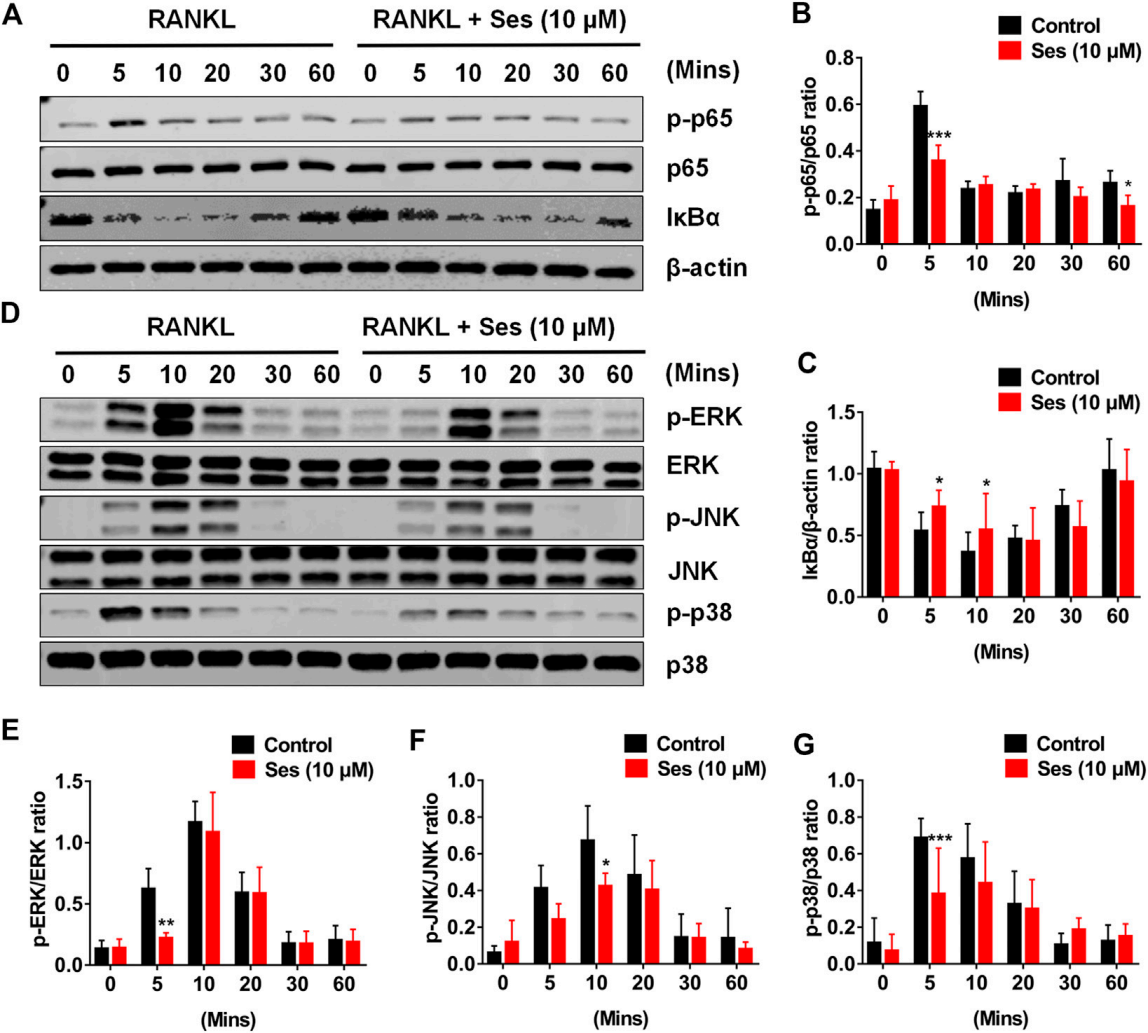
Ses inhibits osteoclast differentiation by inhibiting the activation of NF-κB and the MAPK signaling pathway induced by RANKL. **(A)** Representative Western blot images of the effect of Ses on RANKL-induced IκB-α degradation and p65 protein phosphorylation. **(B**,**C)** The ratio of IκB-α to β-actin and p-p65 to p65 was quantified (*n* = 3). **(D)** Representative Western blot images of the effect of Ses on the RANKL-induced MAPK pathway. Specific antibodies were used to evaluate the protein expression and phosphorylation levels of ERK, JNK and p38. **(E–G)** Quantification of the ratio of phosphorylated ERK, JNK and p38 to the corresponding total protein band. The above data are expressed as the mean ± SD; *p < 0.05, **p < 0.01, and ***p < 0.001. Ses, sesamolin; RANKL, receptor activator of nuclear factor-κB ligand; NF-κB, nuclear factor-κB; MAPKs, mitogen-activated protein kinases.

The NF-κB and MAPK pathways are considered to be the main signal transduction pathways activated during osteoclast formation (Boyce et al., 2015). To further study whether Ses affected the NF-κB and MAPK signaling pathways, thus affecting osteoclast formation, we explored the influence of Ses on these two pathways by Western blot. The experimental data showed that Ses (10 μM) inhibited the degradation of IκB-α within 5 min of RANKL stimulation and lasted for 10 min (Figures 5A,C). After the degradation of IκB-α, p65 is phosphorylated and activated so that it can be transferred from the cytoplasm to the nucleus, thus initiating the transcription of the target gene (Abu-Amer, 2013; Mediero et al., 2013). The results showed that Ses (10 μM) significantly inhibited p65 phosphorylation within 5 min of RANKL stimulation (Figures 5A,B). For the MAPK signaling pathway, the phosphorylation of ERK (Figures 5D,E), JNK (Figures 5D,F) and p38 (Figures 5D,G) was suppressed by Ses. In short, these data indicated that Ses could inhibit RANKL-mediated NF-κB and MAPK signal transduction pathways (Supplementary Figures S3).

### Ses Attenuates the Expression of c-Fos and NFATc1

**FIGURE 6:**
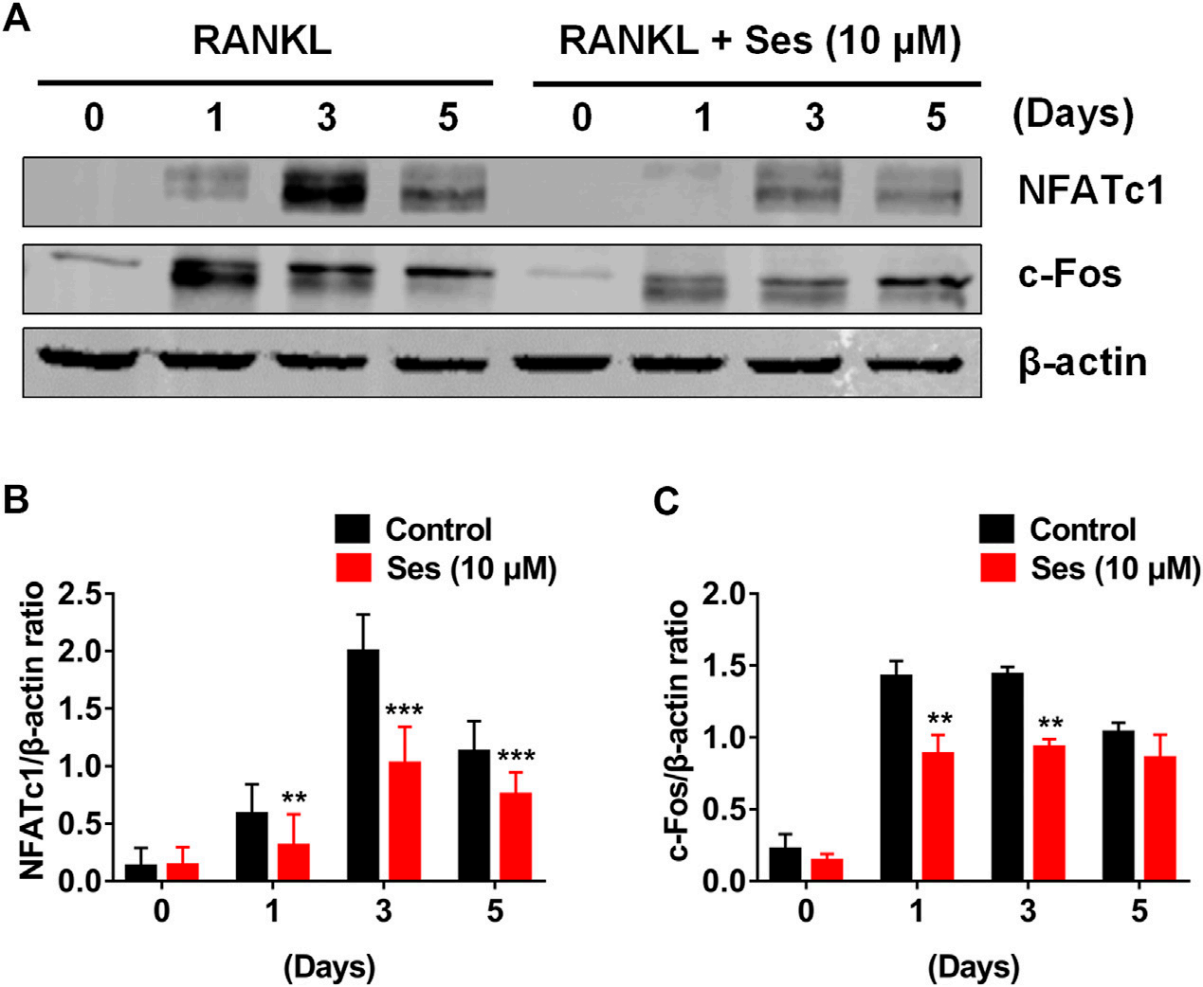
Ses inhibits osteoclast differentiation by inhibiting the expression of c-Fos and NFATc1 induced by RANKL. **(A)** Representative Western blotting of the effect of Ses on the expression of c-Fos and NFATc1 during osteoclast formation. With or without 10 μM Ses treatment, RANKL was used to stimulate BMMs for 0, 1, 3, and 5 days. **(B,C)** Quantitative ratio of NFATc1 and c-Fos to the band strength of β-actin (*n* = 3). The above data are expressed as the mean ± SD; *p < 0.05, **p < 0.01, and ***p < 0.001. Ses, sesamolin; RANKL, receptor activator of nuclear factor-κB ligand; NFATc1, nuclear factor of activated T cells 1; c-Fos, proto-oncogene C-Fos.

c-Fos and NFATc1 are two important transcription factors in the differentiation of osteoclast (Takayanagi et al., 2002). Consistent with the results of qPCR analysis, Western blot results showed that c-Fos and NFATc1 protein expressions were significantly inhibited by Ses (10 μM) (Figures 6A–C). Therefore, Ses treatment not only effectively inhibited the early NF-κB and MAPK signal transduction pathways but also significantly affected the protein expression of c-Fos and NFATc1, thus effectively promoting the anti-osteoclast effect of Ses (Supplementary Figures S4).

### Ses Reduces Bone Loss Induced by OVX in Mice

**FIGURE 7:**
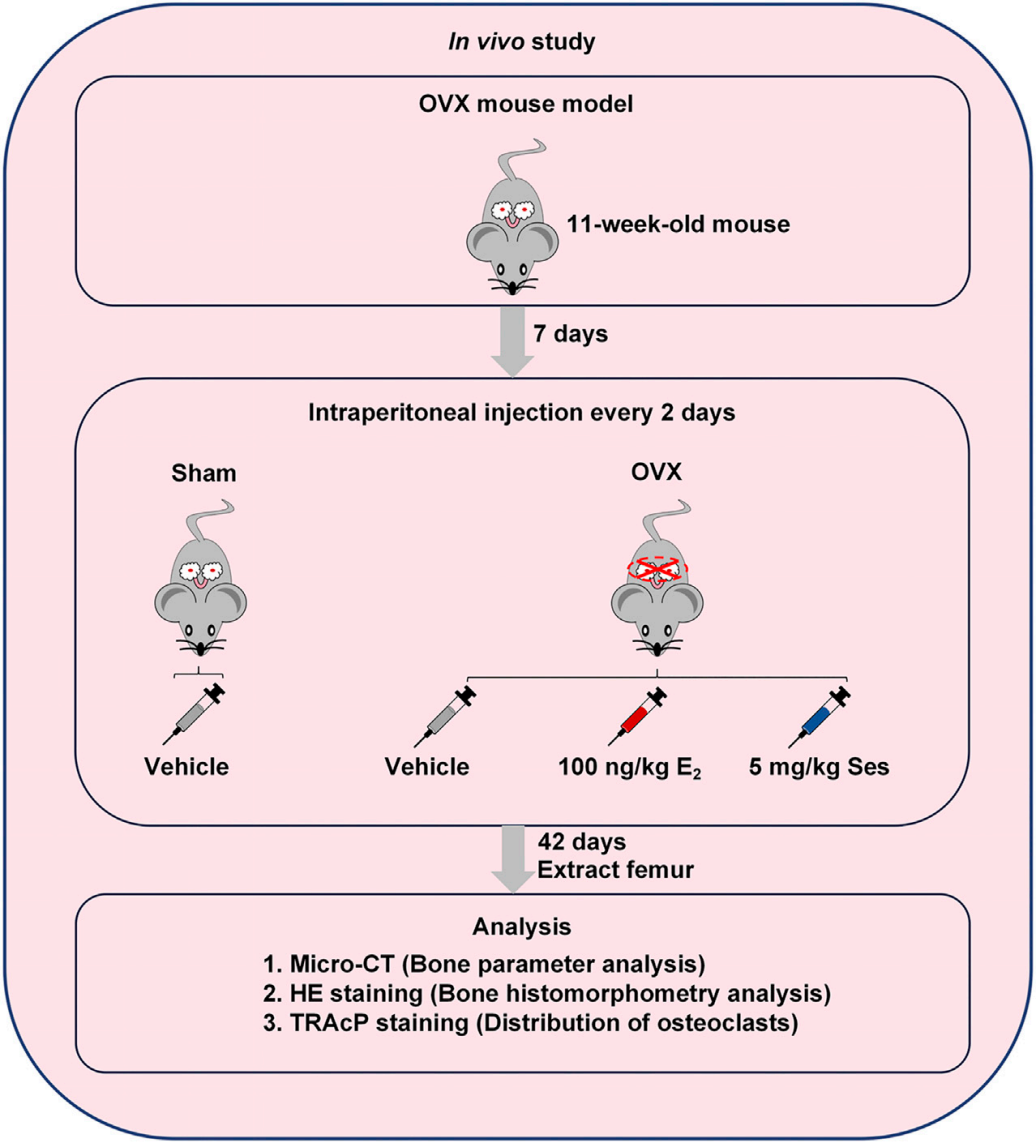
Establishment of an osteoporosis model of OVX mice and an experimental design to evaluate the efficacy of Ses. Ses, sesamolin; Vehicle, 1% DMSO in PBS; E_2_, estrogen; HE, hematoxylin and eosin; TRAcP, tartrate-resistant acid phosphatase.

**FIGURE 8:**
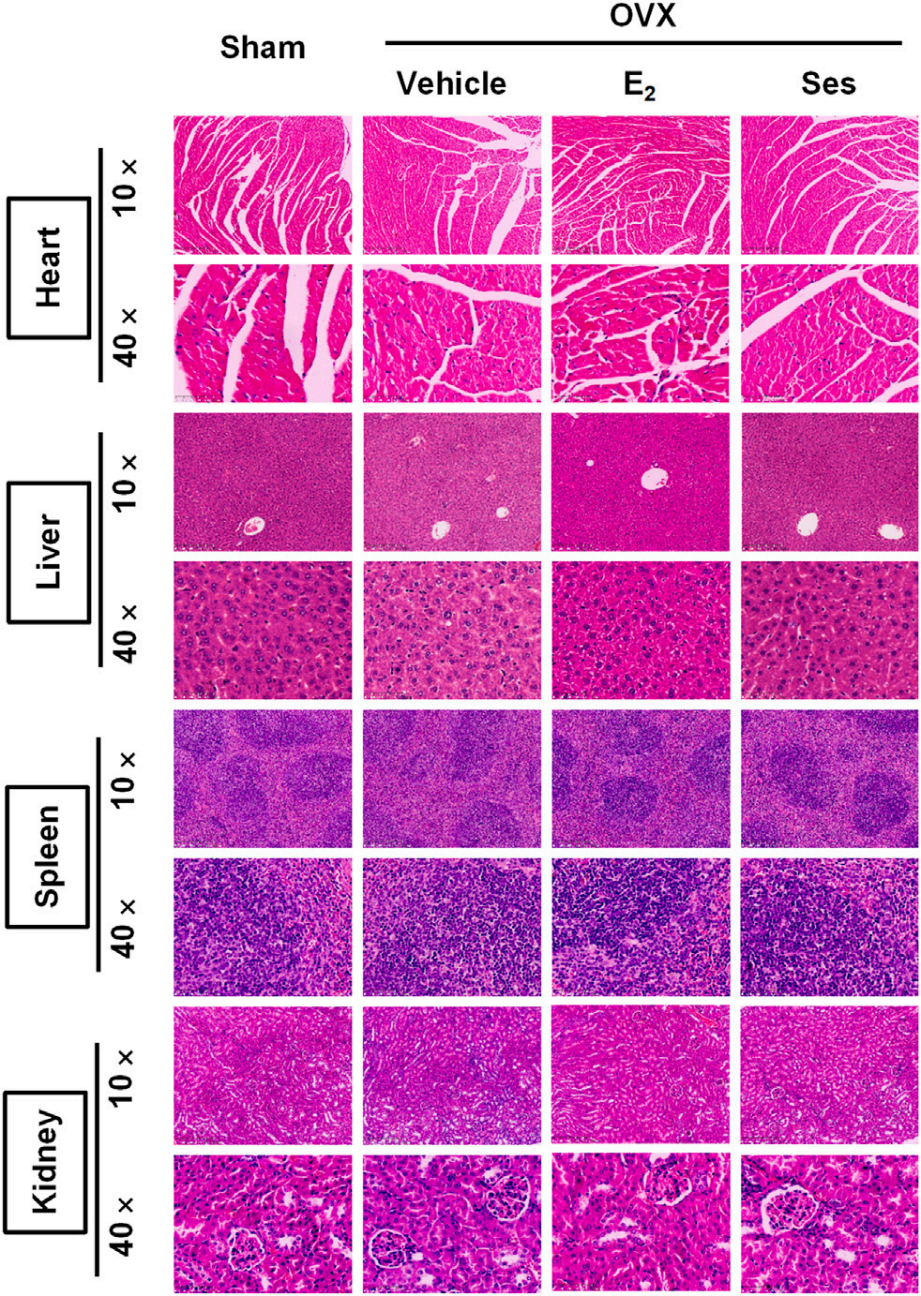
Ses has no obvious toxic effect on the heart, liver, spleen or kidney in ovariectomized mice. Representative images of hematoxylin-eosin staining of the heart, liver, spleen and kidney. Ses, sesamolin; Vehicle, 1% DMSO in PBS; E_2_, estrogen; HE, hematoxylin and eosin.

**FIGURE 9:**
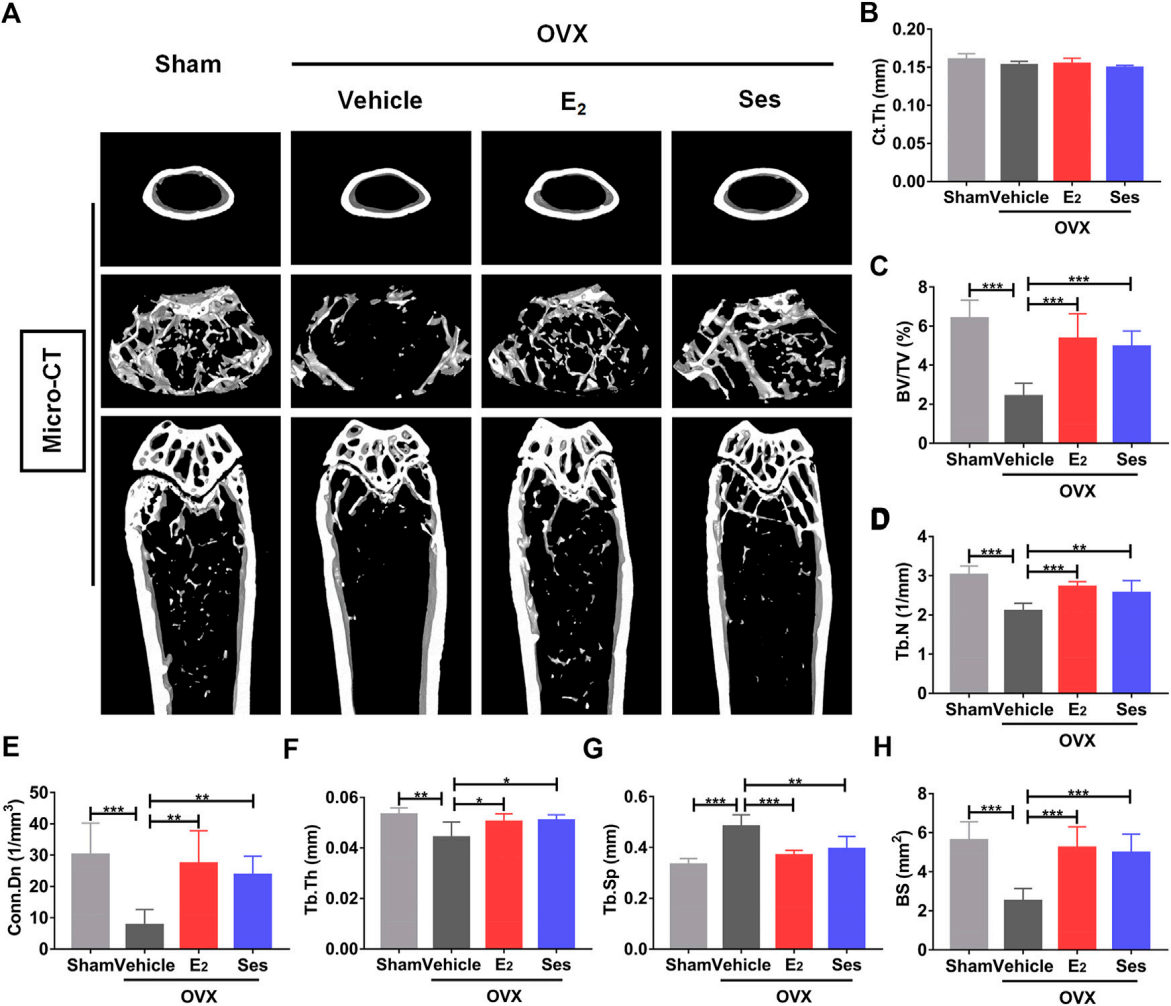
Ses treatment reduces bone loss in ovariectomized (OVX) mice. **(A)** Micro-CT showed that Ses treatment could prevent bone loss. **(B–H)** Quantitative analysis of bone-related parameters, including Ct. Th, BV/TV, Tb.N, Conn. Dn, Tb.Th, Tb. Sp, and BS (*n* = 6). The above data are expressed as the mean ± SD; *p < 0.05, **p < 0.01, and ***p < 0.001. Ses, sesamolin; Vehicle, 1% DMSO in PBS; E_2_, estrogen; Ct. Th, cortical bone thickness; BV/TV, bone volume per tissue volume; Tb.N, trabecular number; Conn. Dn, connectivity density; Tb.Th, trabecular thickness; Tb. Sp, trabecular spacing; BS, bone surface.

**FIGURE 10:**
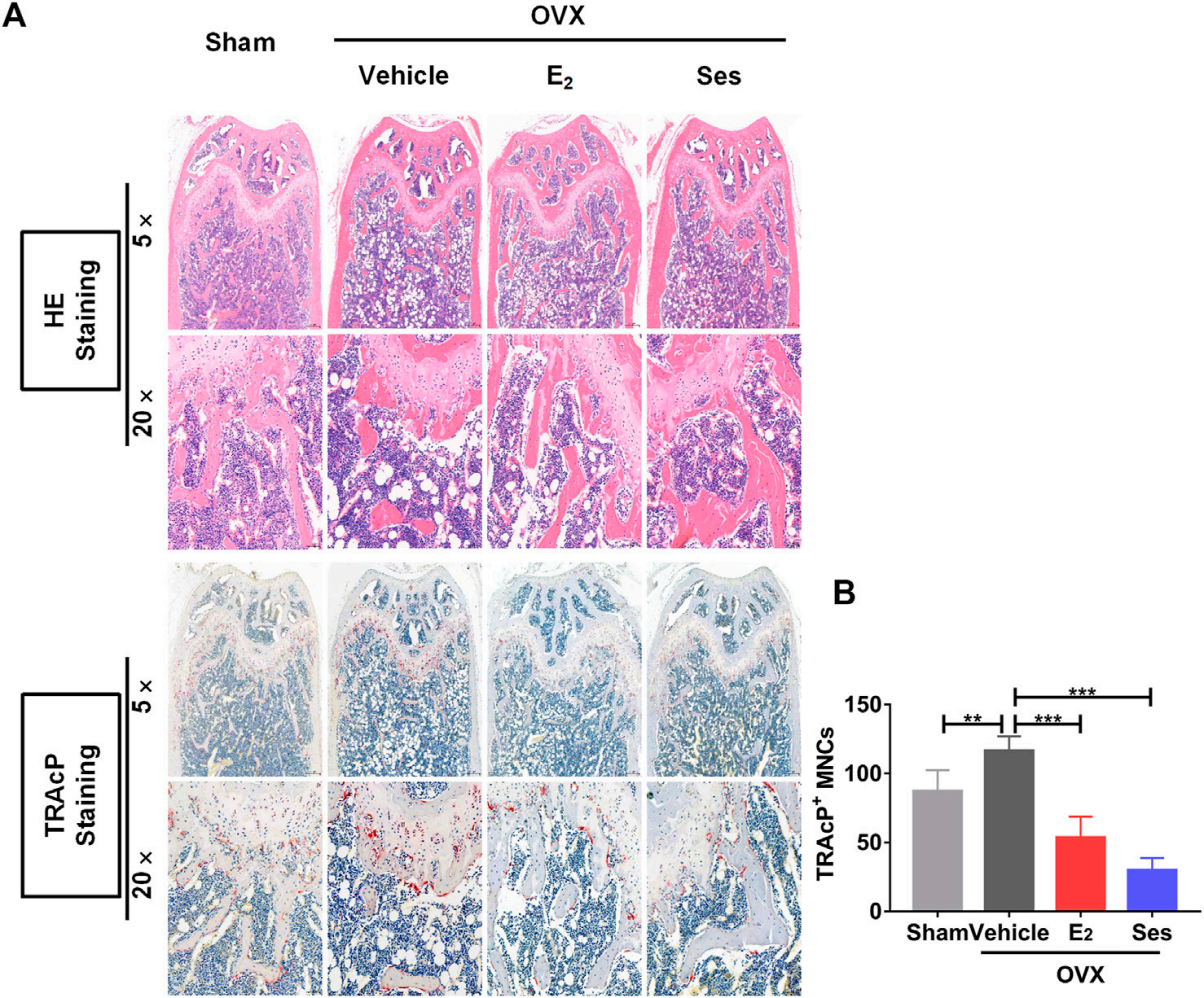
Ses treatment reduces the production of osteoclasts in ovariectomized mice. **(A)** Representative images of histological analysis of femurs stained with HE and TRAcP. **(B)** Quantitative analysis of the number of TRAcP-positive osteoclasts in each group (*n* = 5). The above data are expressed as the mean ± SD; **p < 0.01, and ***p < 0.001. Ses, sesamolin; Vehicle, 1% DMSO in PBS; E_2_, estrogen; HE, hematoxylin and eosin; TRAcP, tartrate-resistant acid phosphatase.

Our results showed that Ses could inhibit the formation and function of osteoclasts *in vitro*. To further explore whether Ses has the potential to prevent diseases, we simulated a model of osteoporosis by OVX in Mice (Figure 7). Mice were injected intraperitoneally with Ses (5 mg/kg), E_2_ (100 ng/kg) or vehicle every two days for 42 days after OVX or sham operations. No deaths or significant adverse effects were recorded during the operations or Ses treatment. HE staining showed that Ses had no obvious toxic effect on the heart, liver, spleen or kidney (Figure 8). Micro-CT results showed that Ses and E_2_ prevented extensive bone loss in the mouse OVX model (Figure 9A). Through quantitative analysis, it was confirmed that, compared with the OVX group, the trabecular parameters BV/TV, Tb.N, Tb.Th, Conn.Dn and BS increased and Tb.Sp decreased in the Ses and E_2_ groups, while there was no significant effect on the cortical bone parameter Ct.th (Figures 9B–H). Histological analysis further confirmed that Ses and E_2_ significantly reduced the OVX-induced bone loss compared with the untreated group. HE staining data further demonstrated that OVX-induced bone loss in mice was improved in the Ses group (Figure 10A). TRAcP staining showed that the number of osteoclasts decreased significantly in the Ses treatment group (Figures 10A,B). Immunohistochemical data showed that Ses treatment group could inhibit the expression of Ctsk in ovariectomized mice, but had no effect on the expression of OCN (Supplementary Figures S2A–C). These results further confirmed the anti-osteoclastogenesis and anti-resorption effects of Ses *in vitro*, and thus protecting OVX mice from osteopenia *in vivo*.

## Discussion

PMOP is the most common bone disease in women. Lack of estrogen leads to the activation of osteoclasts and increased bone resorption of osteoclasts, resulting in bone loss (Miyauchi et al., 2013). At present, there are many adverse reactions associated with the drugs commonly used to treat PMOP. Bisphosphonates may cause gastrointestinal reactions, muscle pain, atrial fibrillation, allergies and other adverse reactions; strontium ranelate increases the risk of venous thromboembolism and allergies; teriparatide may cause headache, nausea, dizziness and other neurological reactions (Rizzoli et al., 2011). To avoid the impact of these side effects, we are urgently looking for an effective drug to treat PMOP without serious side effects. In the present research, we found that Ses inhibited osteoclast formation and bone resorption *in vitro*, which was accompanied by the inhibition of RANKL-induced NF-κB and MAPK signal transduction pathways. Additionally, Ses showed no obvious toxic effects on mouse heart, liver, spleen or kidney *in vivo*, and it was further confirmed that Ses prevented osteoporosis in ovariectomized mice.

First, we examined osteoclast formation and bone resorption, and showed that Ses has the potential to treat osteoclast-related osteoporosis. We also studied the molecular mechanisms by which Ses inhibits osteoclast production and bone resorption, including the NF-κB pathway, the MAPK pathway, c-Fos and NFATc1. Finally, we used the osteoporosis model of ovariectomized mice to evaluate the therapeutic effect and toxicity of Ses *in vivo*.

Osteoclast formation is closely related to RANKL signal transduction. RANKL transduces signals through RANK, and then TRAF6 and cohesive molecules trigger the activation of the NF-κB and MAPK signal transduction pathways (Ono and Nakashima, 2018). NF-κB is a very important factor in the development of osteoclasts. It has been shown that NF-κB double knockout in mice can lead to significant osteopetrosis (Iotsova et al., 1997; Xing et al., 2013; Boyce et al., 2015). In the process of osteoclast differentiation, IκB is phosphorylated in the classical pathway, followed by proteasome degradation, which leads to NF-κB dimer (p50 and p65/RelA) nuclear translocation (Abu-Amer, 2013; Mediero et al., 2013). The binding of RANKL and RANK recruits TRAF6 to activate the MAPK pathway (mainly ERK, JNK and p38) (Soysa et al., 2012). ERK activation is essential in the survival of osteoclasts (Miyazaki et al., 2000). The activation of JNK1 plays a significant role in regulating the formation of osteoclasts, and a lack of JNK1 can lead to a decrease in osteoclast differentiation (David et al., 2002). p38 also plays a key role in osteoclast formation. p38 α regulates the early differentiation of osteoclasts by affecting cell proliferation, and p38 α deficiency leads to an osteopetrotic phenotype in 6-month-old mice (Matsumoto et al., 2000; Cong et al., 2017). In this study, our data showed that Ses treatment could effectively eliminate the degradation of IκB-α and prevent p65 phosphorylation. In addition, we also found that Ses could inhibit the activation of ERK, JNK and p38 signaling. However, the research on the real target of Ses was not performed in this paper, and we do not know whether Ses acts on TRAF6 signals or other early signals. In the next step, we plan to find and verify the real targets of Ses, so as to provide a more mechanistic basis for the development of Ses as a targeted medication for the treatment of PMOP.

NFATc1 is the main regulatory factor in the process of osteoclast differentiation. It activates mature osteoclasts by directly regulating osteoclast-related specific genes (Song et al., 2009). c-Fos is an important component of the transcription factor complex AP-1. c-Fos and NFATc1 collaborate to trigger transcriptional cascades that play a major role in osteoclast differentiation (Grigoriadis et al., 1994; Matsuo et al., 2004; Crotti et al., 2006). Due to the complete loss of osteoclasts, c-Fos-deficient mice can easily develop severe osteopetrosis (Wang et al., 1992). Our Western blot results showed that c-Fos and NFATc1 were significantly inhibited by Ses, which may be a result of the inhibition of NF-κB and MAPK signaling. In addition, our study also found that Ses inhibited the specifically expressed genes in osteoclasts, including *Ctsk*, *Mmp-9*, *Dc-stamp*, *c-Fos* and *NFATc1*, which were directly or indirectly involved in bone resorption (Takayanagi et al., 2002; Kim et al., 2008; Song et al., 2009).

**FIGURE 11:**
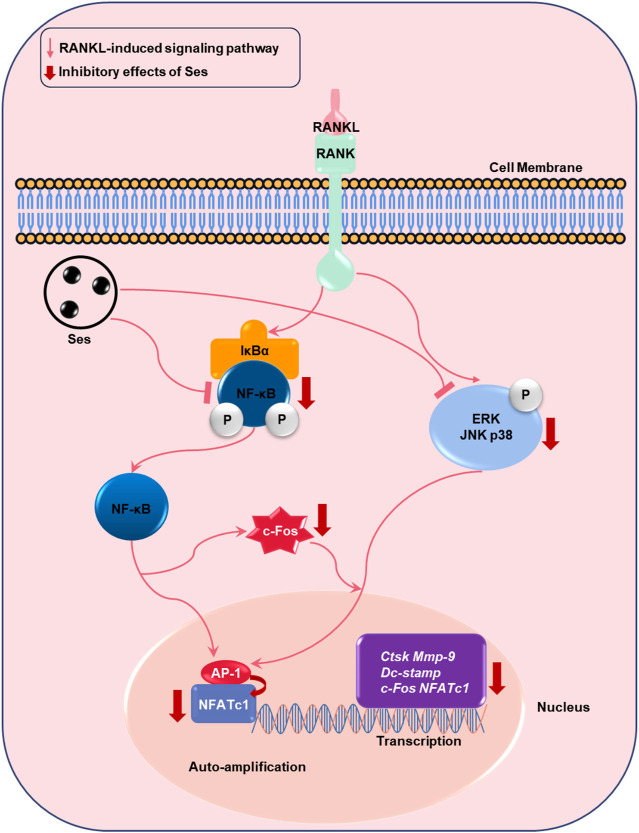
Proposed scheme for Ses inhibition of osteoclast production. When RANKL binds to RANK, both the NF-κB and MAPK pathways are activated, resulting in the amplification of c-Fos and NFATc1. Some genes specifically expressed in osteoclasts are upregulated, such as *Ctsk*, *Mmp-9*, *Dc-stamp*, *c-Fos* and *NFATc1*. These signaling events are mediated by NF-κB and MAPK signaling activation induced by RANKL. Our results demonstrate for the first time that Ses inhibits osteoclast formation by inhibiting the production of NF-κB and MAPK signals induced by RANKL. Ses, sesamolin; RANKL, receptor activator of nuclear facto-κB ligand; NF-κB, nuclear factor-κB; MAPKs, mitogen-activated protein kinases; AP-1, activating protein 1; *c-Fos*, proto-oncogene C-Fos; *Ctsk*, cathepsin K; *Mmp-9*, matrix metalloproteinase-9; *Dc-stamp*, dendritic cell-specific transmembrane protein; *NFATc1*, nuclear factor of activated T cells 1.

In summary, in this research, we demonstrated that Ses could inhibit the activation of MAPK and NF-κB signal transduction pathways induced by RANKL, thereby weakening the expression of c-Fos and NFATc1 transcription factors (Figure 11). These signaling effects were closely related to the inhibitory effect of Ses on osteoclast formation and bone resorption *in vitro*. In addition, our study also found that Ses could effectively prevent bone loss in ovariectomized mice and showed no significant adverse effects on the heart, liver, spleen or kidney. These results provide promising evidence for Ses as a candidate medication for the treatment of PMOP.

## Data Availability

The original contributions presented in the study are included in the article/Supplementary Material, further inquiries can be directed to the corresponding authors.

## Supplementary Material

Figure S1 Ses inhibits the expression of osteoclast-specific genes in vivo. (A-B) The expression levels of the osteoclast-related specific genes Ctsk and Mmp-9 were analyzed by qPCR. The above data are expressed as the mean ± SD; *p < 0.05 and ***p < 0.001. Ses, sesamolin; Vehicle, 1% DMSO in PBS; E2, estogen; Ctsk, cathepsin K, Mmp-9, matrix metalloproteinase-9.

Figure S2 Ses inhibits the expression of Ctsk in ovariectomized mice, but has no effect on the expression of OCN. (A) The representative images of Ctsk and OCN immunohistochemical staining in mice in each group. (B) Quantitative analysis of the number of Ctsk-positive osteoclasts in each group (n = 3). (C) Quantitative analysis of OCN positive area in each group (n = 3). The above data are expressed as the mean ±; **p < 0.01. Ses, sesamolin; Vehicle, 1% DMSO in PBS; E2, estrogen; Ctsk, cathepsin K, OCN, osteocalcin.

Figure S3 The original Western Blot images of p-p65, p65, IκB-α, β-actin, p-ERK, ERK, p-JNK, JNK, p-p38and p38.

Figure S4 The original Western Blot images of NFATc1, c-Fos and β-actin.
