# Supplementary figures and images for: Sesamolin Protects Mice From Ovariectomized Bone Loss by Inhibiting Osteoclastogenesis and RANKL-Mediated NF-κB and MAPK Signaling Pathways
Source: Front Pharmacol. 2021 Jun 14;12:664697. doi: 10.3389/fphar.2021.664697 (PMC8237092; doi:10.3389/fphar.2021.664697)

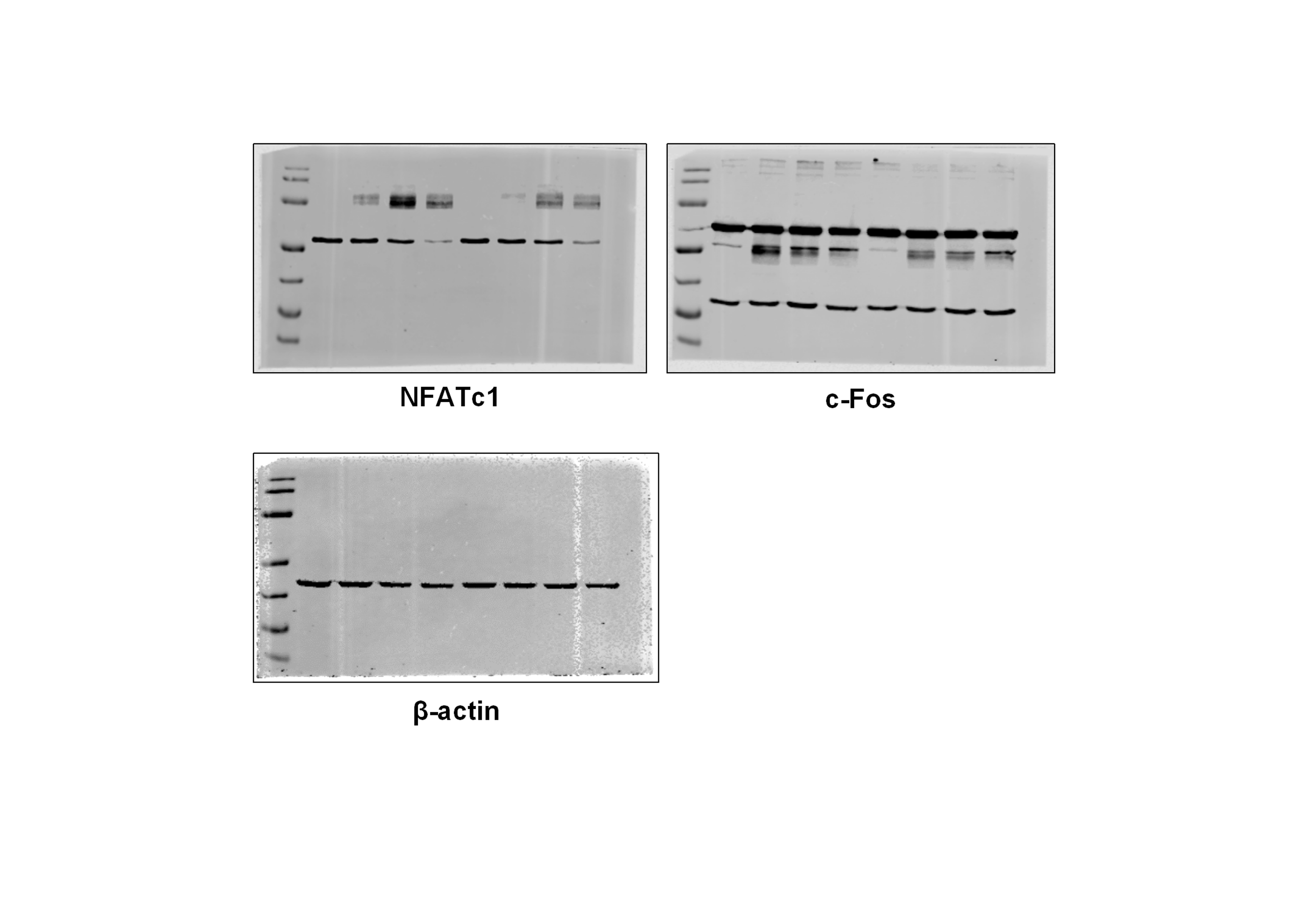

Supplement: Figure S1 — Ses inhibits the expression of osteoclast-specific genes in vivo. (A-B) The expression levels of the osteoclast-related specific genes Ctsk and Mmp-9 were analyzed by qPCR. The above data are expressed as the mean ± SD; *p < 0.05 and ***p < 0.001. Ses, sesamolin; Vehicle, 1% DMSO in PBS; E2, estogen; Ctsk, cathepsin K, Mmp-9, matrix metalloproteinase-9. [file image1.tif]

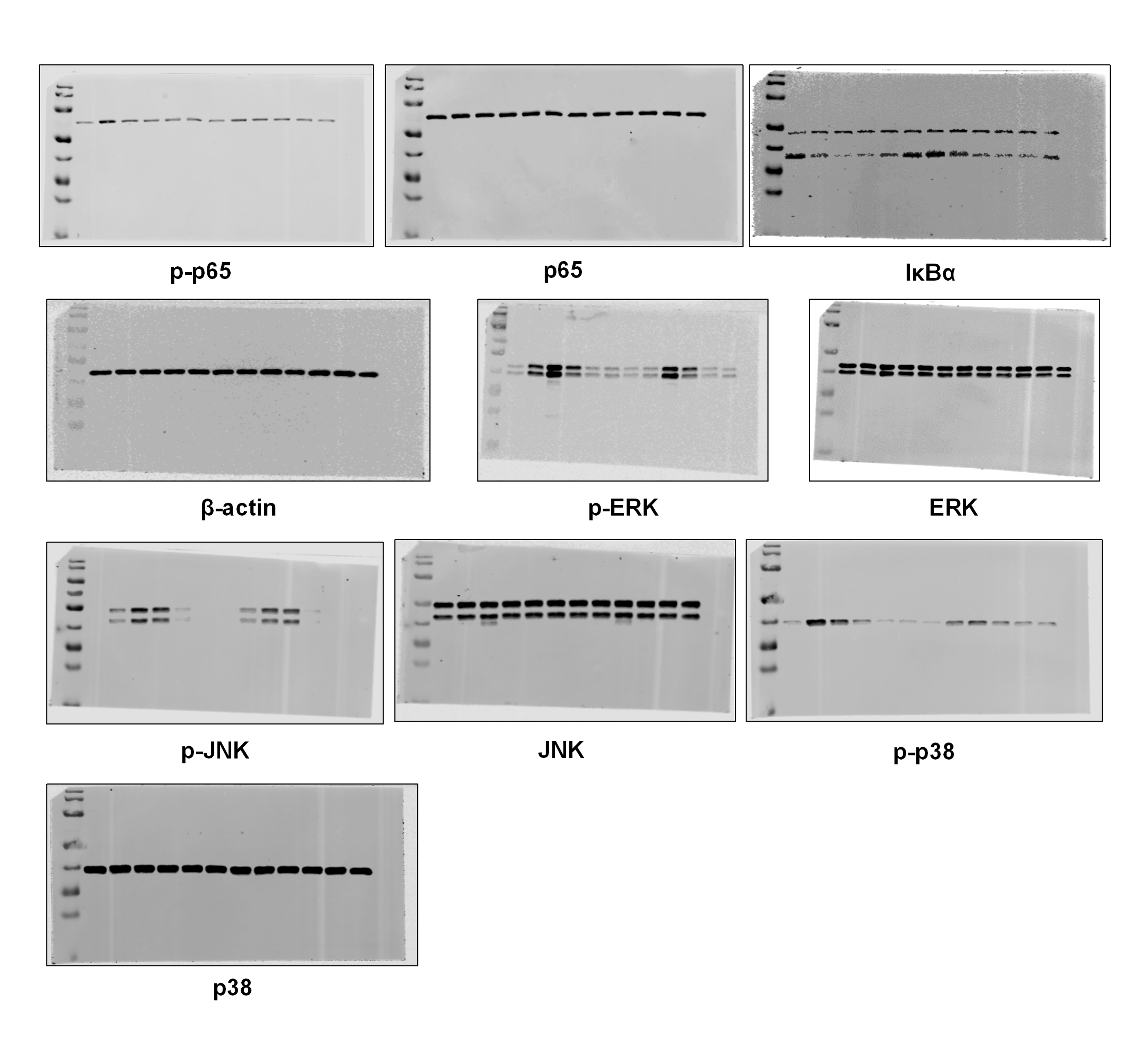

Supplement: Figure S2 — Ses inhibits the expression of Ctsk in ovariectomized mice, but has no effect on the expression of OCN. (A) The representative images of Ctsk and OCN immunohistochemical staining in mice in each group. (B) Quantitative analysis of the number of Ctsk-positive osteoclasts in each group (n = 3). (C) Quantitative analysis of OCN positive area in each group (n = 3). The above data are expressed as the mean ±; **p < 0.01. Ses, sesamolin; Vehicle, 1% DMSO in PBS; E2, estrogen; Ctsk, cathepsin K, OCN, osteocalcin. [file image2.tif]

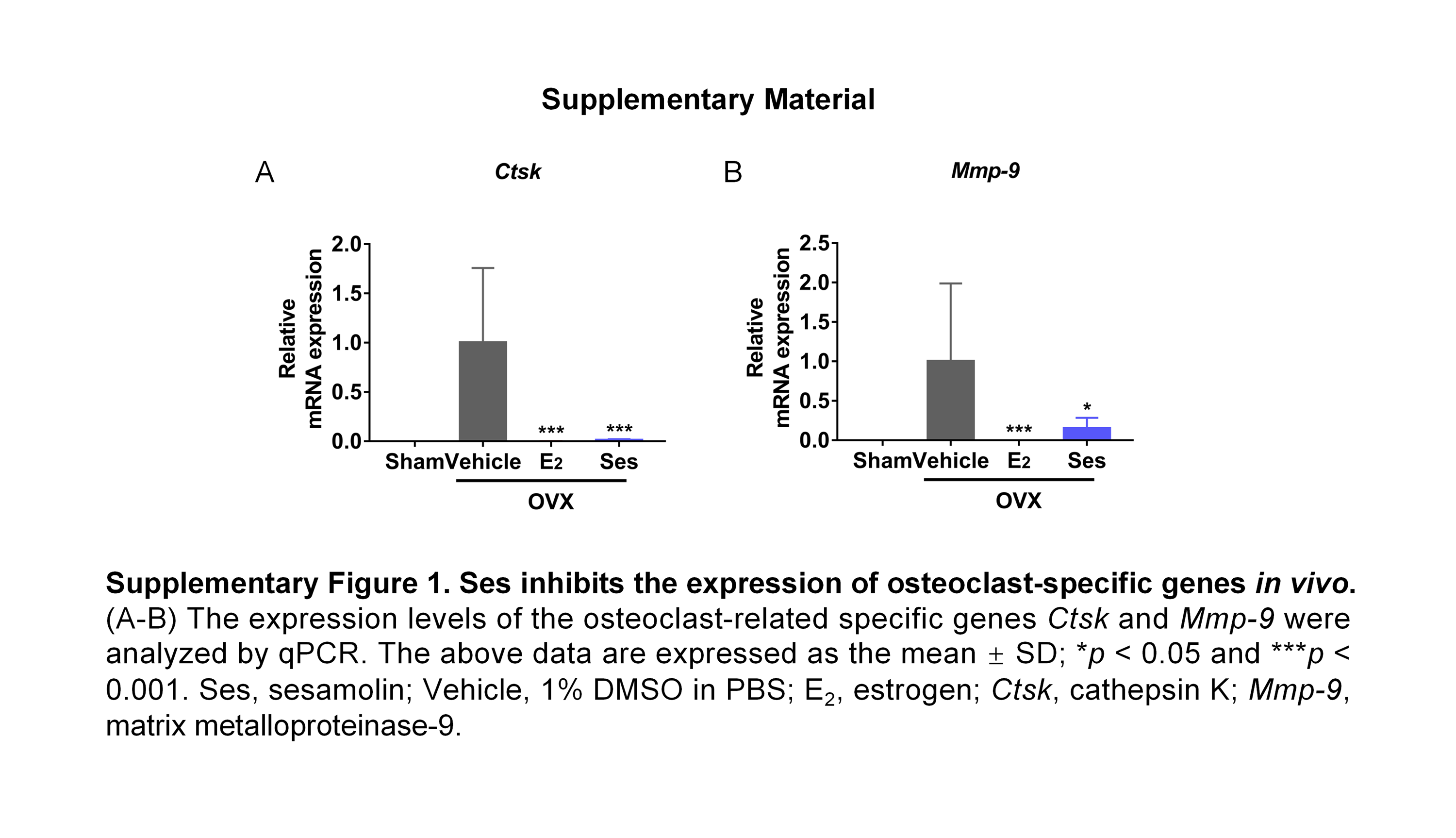

Supplement: Figure S3 — The original Western Blot images of p-p65, p65, IκB-α, β-actin, p-ERK, ERK, p-JNK, JNK, p-p38and p38. [file image3.tif]

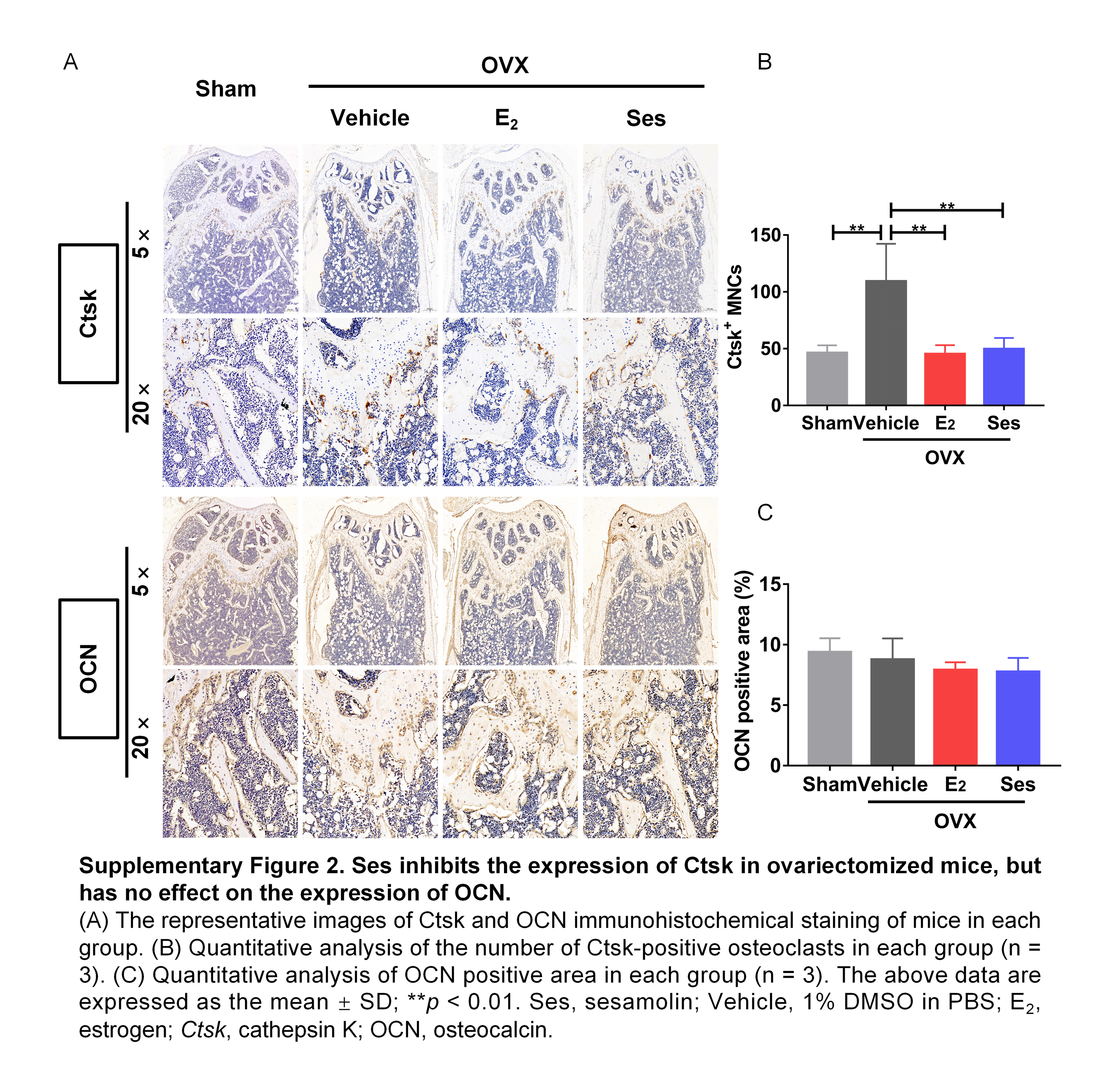

Supplement: Figure S4 — The original Western Blot images of NFATc1, c-Fos and β-actin. [file image4.tif]
